# Associations between Tumor Necrosis Factor-α Polymorphisms and Risk of Psoriasis: A Meta-Analysis

**DOI:** 10.1371/journal.pone.0068827

**Published:** 2013-12-04

**Authors:** Le Zhuang, Weiyuan Ma, Daxing Cai, Hua Zhong, Qing Sun

**Affiliations:** Department of Dermatology, Qilu Hospital, Shandong University, Jinan, China; Tor Vergata University of Rome, Italy

## Abstract

**Background:**

Tumor necrosis factor-α (TNF-α) may play an important role in the recalcitrant inflammatory and hyperproliferative dermatosis of psoriasis, and there may be a relationship between TNF-α polymorphisms and psoriasis risk.

**Methods:**

We performed a meta-analysis to evaluate the associations between TNF-α polymorphisms and psoriasis. Electronic searches of Pubmed, Embase, and Web of Science were performed for all publications on the associations between TNF-α polymorphisms and psoriasis through September 26, 2012. The pooled odds ratios (ORs) with their 95% confidence interval (95%CIs) were calculated to assess the associations.

**Results:**

Sixteen case-control studies with a total of 2,253 psoriasis cases and 1,947 controls on TNF-α 308 G/A polymorphism and fourteen studies on TNF-α 238 G/A polymorphism with 2,104 cases and 1,838 controls were finally included into the meta-analysis. Overall, TNF-α 308 G/A polymorphism was significantly associated with decreased risk of psoriasis under three genetic comparison models (for A versus G: fixed-effects OR 0.71, 95%CI 0.62-0.82, P < 0.001; for AG versus GG: fixed-effects OR 0.67, 95%CI 0.57-0.78, P < 0.001; for AA/AG versus GG: fixed-effects OR 0.67, 95%CI 0.58-0.78, P < 0.001). In addition, TNF-α 238 G/A polymorphism was associated with increased risk of psoriasis under three genetic models (for A versus G: fixed-effects OR 2.46, 95%CI 2.04-2.96, P < 0.001; for AG versus GG: fixed-effects OR 2.69, 95%CI 2.20-3.28, P < 0.001; for AA/AG versus GG: fixed-effects OR 2.68, 95%CI 2.20-3.26, P < 0.001). Subgroup analysis by ethnicity identified a significant association between TNF-α 308 G/A polymorphism and decreased risk of psoriasis in both Caucasians and Asians and a significant association between TNF-α 238 G/A polymorphism and increased risk of psoriasis in Caucasians.

**Conclusions:**

The meta-analysis suggests that TNF-α 308 G/A polymorphism is associated with decreased risk of psoriasis, while TNF-α 238 G/A is associated with increased risk of psoriasis.

## Introduction

Psoriasis is a very common inflammatory skin disease which affects about 3% total populations in the world [1,2]. Currently, the pathogenesis of psoriasis is still unclear and need further studies [1,3,4]. Previous studies suggest psoriatic skin is characterized by a large number of inflammation and epidermal proliferation markers, and large numbers of immune cells exist in the psoriatic skin which can produce many cytokines and inflammatory molecules [1,4]. Evidence form previous genome-wide association studies is for that genetic susceptibility factors also play an important role in the inflammatory and immune actions of psoriatic skin, and one candidate gene is the tumor necrosis factor-α (TNF-α) gene [3,5-7]. TNF-α is an important inflammatory mediator and its expression has been shown to be involved in the development of psoriatic lesions [8,9]. There are several common single nucleotide polymorphisms (SNP) in the TNF-α gene, including −238 (rs361525), −308 (rs1800629), and −857 (rs1799724) positions, which can regulate the transcription and production of TNF-α, and the most studied polymorphisms are a G to A transition in the promoter at position –308 and another G to A transition in the promoter at position –238 [10]. Although possible associations of the TNF-α polymorphisms with psoriasis were reported, and many case-control studies were further performed to identify the association, it was still unknown whether there were significant associations of TNF-α 308 G/A and 238 G/A polymorphisms with psoriasis risk [11-20]. Therefore, we conducted a systematic review and meta-analysis of previous published case-control studies to comprehensively evaluate the associations of TNF-α 308 G/A and 238 G/A polymorphisms with psoriasis risk. 

## Materials and Methods

### Identification and eligibility of relevant studies

Electronic searches of Pubmed, Embase, and Web of Science were performed for all publications on the associations of TNF-α 308 G/A and 238 G/A polymorphisms with psoriasis risk through September 26, 2012. We used the keywords and subject terms: (“psoriasis” or “psoriatic”) and (“polymorphism” or “variant” or “genotype” or “polymorphism”) and (“tumor necrosis factor” or “TNF 238 G/A” or “TNF 308 G/A” or “TNF-α” or “rs1800629”). There was no language limitation in the literature search. All eligible studies were retrieved, and their bibliographies were checked for other relevant publications. The following criteria were used to select the eligible studies: (1) case-control studies involving the associations of TNF-α 308 G/A and 238 G/A polymorphisms with psoriasis risk; (2) reporting genotype frequencies of TNF-α 308 G/A polymorphism for estimating an odds ratio (OR) with 95% confidence interval (95%CI) (3) Confirmation of Hardy-Weinberg equilibrium (HWE) in the controls. If two or more studies reported the same patients populations, only the most recent or complete study was included into this meta-analysis.

### Data extraction

The final eligible articles selected for meta-analysis were carefully evaluated independently by two reviewers, and discrepancies were adjudicated by the consensus among all reviewers. Data retrieved from the reports included first author’s name, publication year, ethnicity of study population (categorized as Caucasians and Asians), study-design (sources of controls), genotyping method, types of cancer, number of cases and controls, and genotype frequencies of TNF-α 308 G/A or 238 G/A polymorphisms in cases and controls.

### Statistical methods

HWE in the controls was tested by a Chi-square test which compared the observed and expected genotype frequencies of the controls [21]. The pooled ORs with the corresponding 95%CIs were calculated by meta-analysis to evaluate the associations of TNF-α 308 G/A and 238 G/A polymorphisms with psoriasis risk, and an OR greater than 1 indicated a increased risk of psoriasis. The statistical significance of the summary OR was determined using the Z-test and a P value of less than 0.05 was considered significant. To get a more comprehensive assessment of associations of TNF-α 308 G/A and 238 G/A polymorphisms with psoriasis risk, five comparison model were used: the allele comparison model (A versus G), the homozygote comparison model (AA versus GG), the heterozygote comparison model (AG versus GG), the dominant genetic model (AA/AG versus GG), and the recessive genetic model (AA versus AG/GG). The I^2^ statistic to quantify the proportion of the total variation due to heterogeneity were calculated, and a I^2^ value of more than 50% was interpreted as significant heterogeneity among studies [22]. When the effects were assumed to be homogenous, the fixed-effects model was used (Mantel-Haenszel method) [23]. If obvious heterogeneity was present, the random-effects model was used (DerSimonian-Laird method) [24]. To validate the credibility of outcomes in this meta-analysis, sensitivity analysis was performed by sequential omission of individual studies [25]. Potential publication bias was assessed by visual inspection of the funnel plots, in which the standard error of logOR of each study was plotted against its logOR, and an asymmetric plot suggested possible publication bias. In addition, We also performed Egger linear regression test at the P < 0.05 level of significance to assess the funnel-plot’s asymmetry [26]. All analyses were conducted using STATA (Version 11, StataCorp, College Station, TX), and P values were two sided.

## Results

### Characteristics of studies

The study selection was shown in Figure S1. According to eligibility criteria and exclusion criteria, seventeen individual case-control studies were faintly identified [11-20,27-33]. Sixteen case-control studies with a total of 2,253 psoriasis cases and 1,947 controls on TNF-α 308 G/A polymorphism [11-20,27-32] and fourteen studies on TNF-α 238 G/A polymorphism with 2,104 cases and 1,838 controls [11-17,19,28-33] were finally included into the meta-analysis. These studies were published between 1999 and 2012, and most study designs were hospital-based case-control studies [11-20,27-33]. For TNF-α 308 G/A polymorphism, there were a total of 12 studies from Caucasian population [11-13,15,17,18,20,27-29,31,32], 3 studies from Asian population [16,19,30], and only one study from the other populations [14]. For TNF-α 238 G/A polymorphism, there were a total of 10 studies from Caucasian population [11–13,15,18,28,29,31–33], 3 studies from Asian population [16,19,30], and only one study from the other populations [14]. The cases in 13 studies were selected from patients with psoriasis [11-14,16-20,27,30,31,33], while the other 4 studies were from patients with psoriatic arthritis [15,28,29,32]. All 17 publications were published in English, and the most common method for testing genotype frequencies of TNF-α polymorphisms was polymerase chain reaction-restriction fragment length polymorphism (PCR-RFLP). The genotype distributions of TNF-α 308 G/A and 238 G/A polymorphisms in the controls of all studies were in agreement with HWE (P > 0.05). 

### Meta-analysis

**Figure 1:**
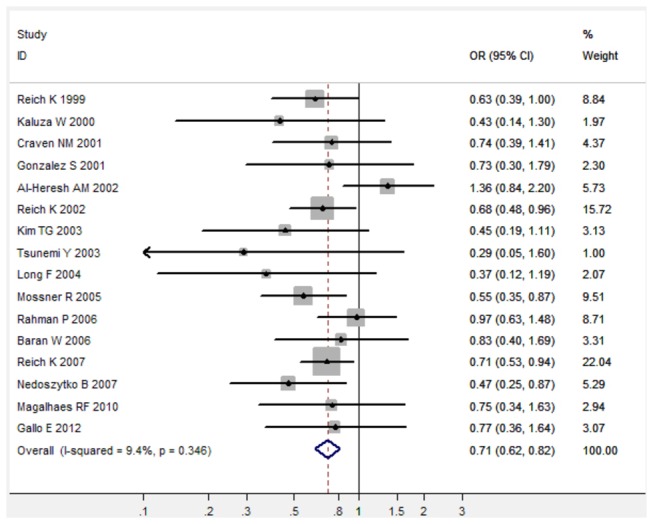
Forest plot in the meta-analysis of TNF-α 308 G/A polymorphism and psoriasis risk under the allele comparison model (A versus G).

**Figure 2:**
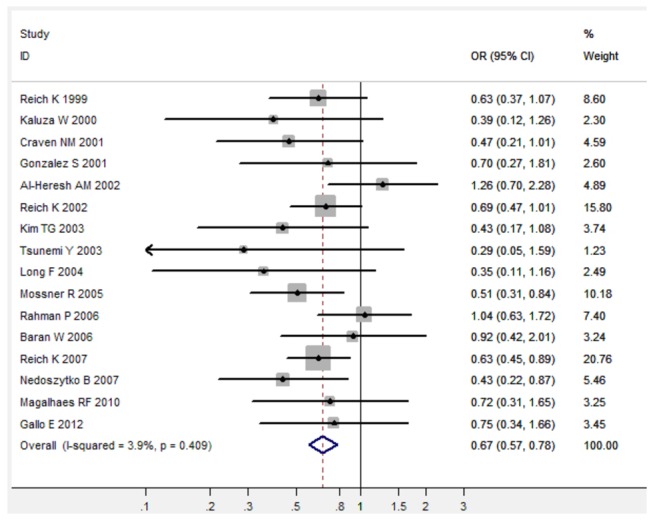
Forest plot in the meta-analysis of TNF-α 308 G/A polymorphism and psoriasis risk under the allele comparison model under the heterozygote comparison model (AG versus GG).

**Figure 3:**
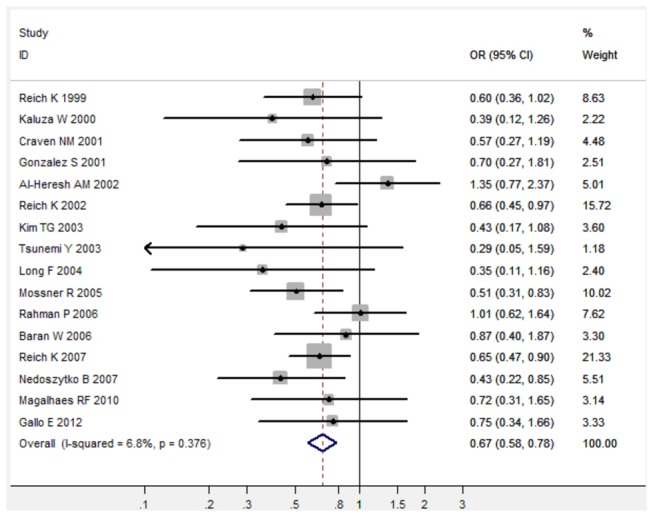
Forest plot in the meta-analysis of TNF-α 308 G/A polymorphism and psoriasis risk under the dominant genetic model ( **AA**/**AG** versus GG).

In the meta-analysis of total 16 studies, there was no obvious between-study heterogeneity in all five comparison models (All I^2^ < 50%), thus the fixed-effects model was used to calculated pooled ORs with the corresponding 95%CIs (Table 1). Overall, TNF-α 308 G/A polymorphism was significantly associated with decreased risk of psoriasis under three genetic comparison models (For A versus G: fixed-effects OR 0.71, 95%CI 0.62-0.82, P < 0.001; for AG versus GG: fixed-effects OR 0.67, 95%CI 0.57-0.78, P < 0.001; for AA/AG versus GG: fixed-effects OR 0.67, 95%CI 0.58-0.78, P < 0.001) when all 16 studies were pooled into the meta-analysis (Figure 1, Figure 2, and Figure 3). In the subgroup analysis of 12 studies from Caucasian populations, there was also no obvious between-study heterogeneity in all five comparison models (All I^2^ < 50%), thus the fixed-effects model was used to calculated pooled ORs with the corresponding 95%CIs (Table 1). TNF-α 308 G/A polymorphism was significantly associated with decreased risk of psoriasis under three genetic comparison models in Caucasian population (Table 1). In the subgroup analysis of 3 studies from Asian populations, there was also no between-study heterogeneity in those three comparison models (All I^2^ = 0.0%), thus the fixed-effects model was used to calculated pooled ORs with the corresponding 95%CIs (Table 1). TNF-α 308 G/A polymorphism was significantly associated with decreased risk of psoriasis under three genetic comparison models in Asian populations when those 3 studies were pooled into the meta-analysis (Table 1). 

**Table 1:** Meta-analysis of the association between TNF-α 308 G/A polymorphism and psoriasis risk.

**Groups**	**Studies**	**Subjects (Cases/Controls)**	**OR [95%CI]**	**P value **	**I^2^ value**
Total studies					
A vs. G	16	2,253/1,947	0.71[0.62-0.82]	<0.001	9.4%
AG vs. GG	16	2,253/1,947	0.67[0.57-0.78]	<0.001	3.9%
AA vs. GG	16	2,253/1,947	0.77[0.45-1.33]	0.345	0.0%
AG/AA vs. GG	16	2,253/1,947	0.67[0.58-0.78]	<0.001	6.8%
AA vs. AG/GG	16	2,253/1,947	0.83[0.48-1.43]	0.498	0.0%
Caucasian population					
A vs. G	12	1,846/1,547	0.73[0.64-0.84]	<0.001	16.6%
AG vs. GG	12	1,846/1,547	0.69[0.59-0.81]	<0.001	12.6%
AA vs. GG	12	1,846/1,547	0.77[0.45-1.33]	0.345	0.0%
AG/AA vs. GG	12	1,846/1,547	0.69[0.59-0.81]	<0.001	15.7%
AA vs. AG/GG	12	1,846/1,547	0.83[0.48-1.43]	0.498	0.0%
Asian population					
A vs. G	3	343/303	0.40[0.21-0.77]	0.006	0.0%
AG vs. GG	3	343/303	0.38[0.20-0.75]	0.005	0.0%
AG/AA vs. GG	3	343/303	0.38[0.20-0.75]	0.005	0.0%

(**Abbreviations**: OR, odds ratio; 95%CI, 95% confidence interval; TNF-α, Tumor necrosis factor-α)

**Figure 4:**
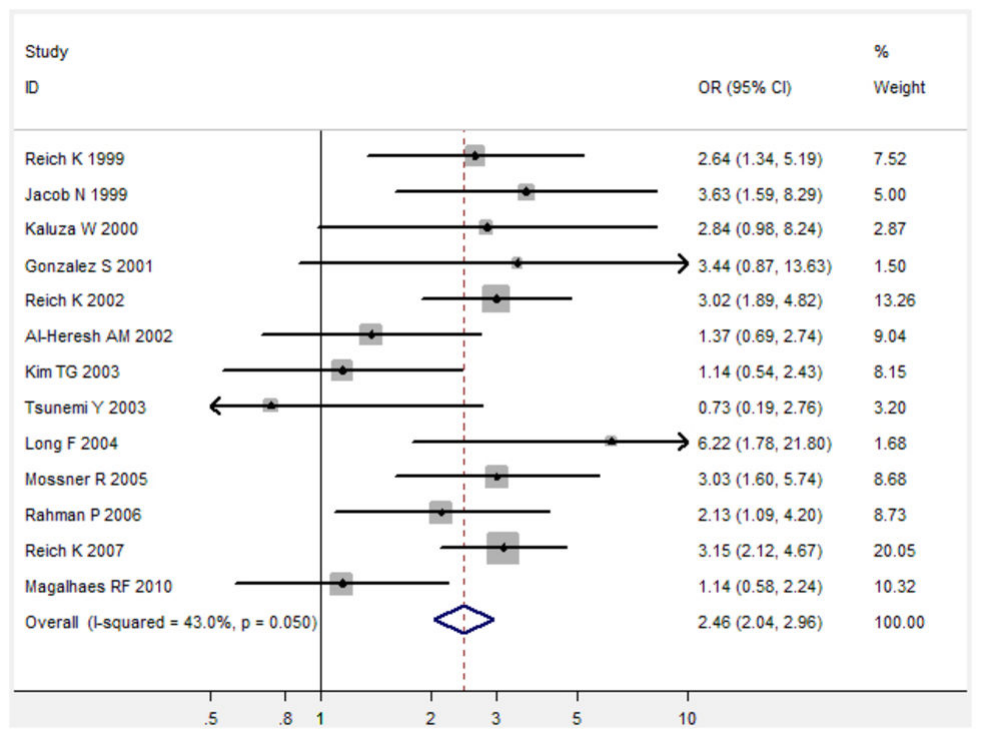
Forest plot in the meta-analysis of TNF-α 238 G/A polymorphism and psoriasis risk under the allele comparison model (A versus G).

**Figure 5:**
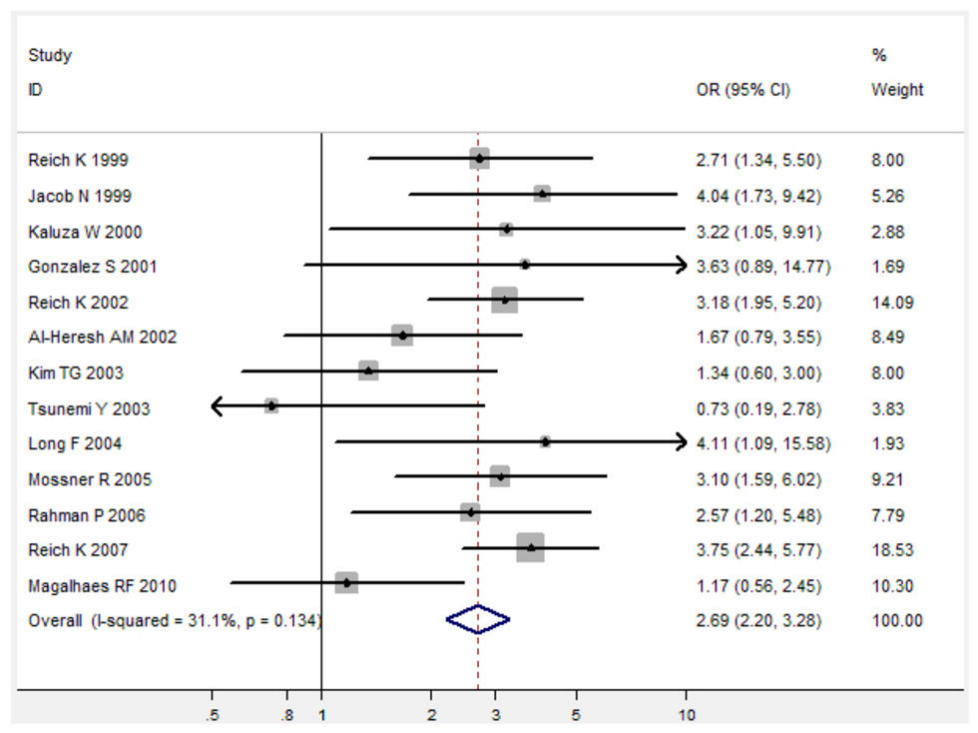
Forest plot in the meta-analysis of TNF-α 238 G/A polymorphism and psoriasis risk under the allele comparison model under the heterozygote comparison model (AG versus GG).

**Figure 6:**
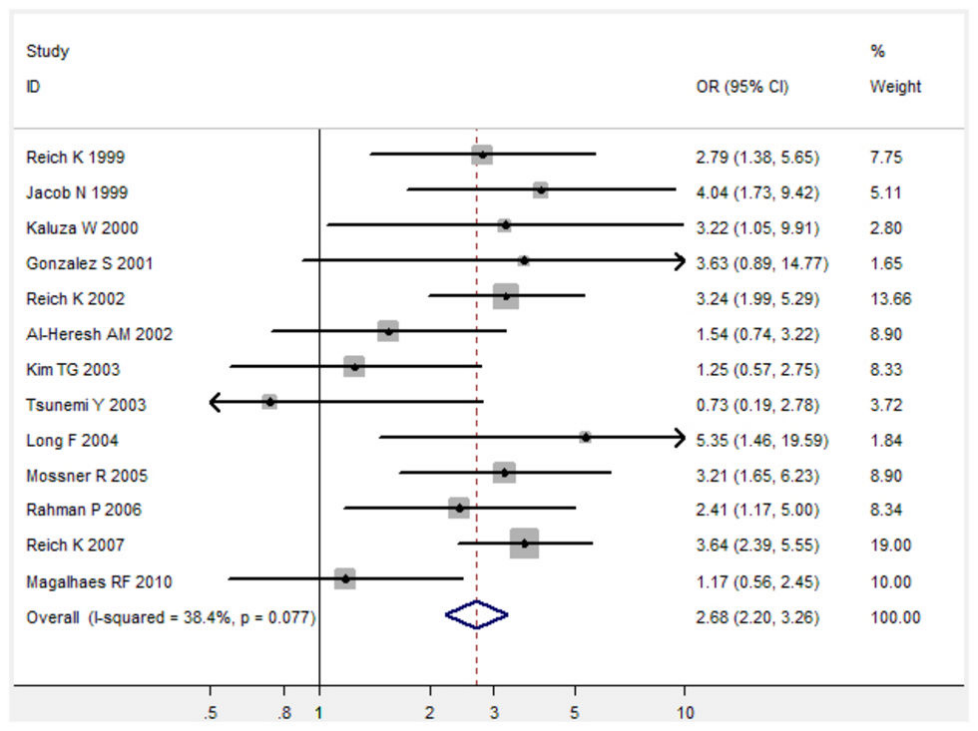
Forest plot in the meta-analysis of TNF-α 238 G/A polymorphism and psoriasis risk under the dominant genetic model ( **AA**/**AG** versus GG).

For TNF-α 238 G/A polymorphism, TNF-α 238 G/A polymorphism was associated with increased risk of psoriasis under three genetic models (For A versus G: fixed-effects OR 2.46, 95%CI 2.04-2.96, P < 0.001; for AG versus GG: fixed-effects OR 2.69, 95%CI 2.20-3.28, P < 0.001; for AA/AG versus GG: fixed-effects OR 2.68, 95%CI 2.20-3.26, P < 0.001) when all 14 studies were pooled into the meta-analysis (Figure 4, Figure 5, and Figure 6, Table 2). Subgroup analysis by ethnicity further identified a significant association between TNF-α 238 G/A polymorphism and increased risk of psoriasis in Caucasians (Table 2).

**Table 2:** Meta-analysis of the association between TNF-α 238 G/A polymorphism and psoriasis risk.

**Groups**	**Studies**	**Subjects (Cases/Controls)**	**OR [95%CI]**	**P value**	**I^2^ value**
Total studies					
A vs. G	14	2,104/1,838	2.46[2.04-2.96]	<0.001	43.0%
AG vs. GG	14	2,253/1,947	2.69[2.20-3.28]	<0.001	31.1%
AA vs. GG	14	2,253/1,947	1.87[0.79-4.44]	0.154	0.0%
AG/AA vs. GG	14	2,253/1,947	2.68[2.20-3.26]	<0.001	38.4%
AA vs. AG/GG	14	2,253/1,947	1.64[0.69-3.89]	0.265	0.0%
Caucasian population					
A vs. G	10	1,692/1,465	2.76[2.24-3.41]	<0.001	0.0%
AG vs. GG	10	1,692/1,465	3.10[2.48-3.88]	<0.001	0.0%
AA vs. GG	10	1,692/1,465	1.71[0.63-4.65]	0.294	0.0%
AG/AA vs. GG	10	1,692/1,465	3.06[2.46-3.82]	<0.001	0.0%
AA vs. AG/GG	10	1,692/1,465	1.46[0.54-3.97]	0.460	0.0%
Asian population					
A vs. G	3	343/303	1.70[0.98-2.93]	0.058	70.3%
AG vs. GG	3	343/303	1.56[0.86-2.84]	0.144	41.4%
AA vs. GG	3	343/303	2.45[0.44-13.57]	0.305	46.2%
AG/AA vs. GG	3	343/303	1.64[0.58-4.63]	0.347	60.6%
AA vs. AG/GG	3	343/303	1.88[0.10-34.58]	0.672	43.6%

(**Abbreviations**: OR, odds ratio; 95%CI, 95% confidence interval; TNF-α, Tumor necrosis factor-α)

### Publication bias

**Figure 7:**
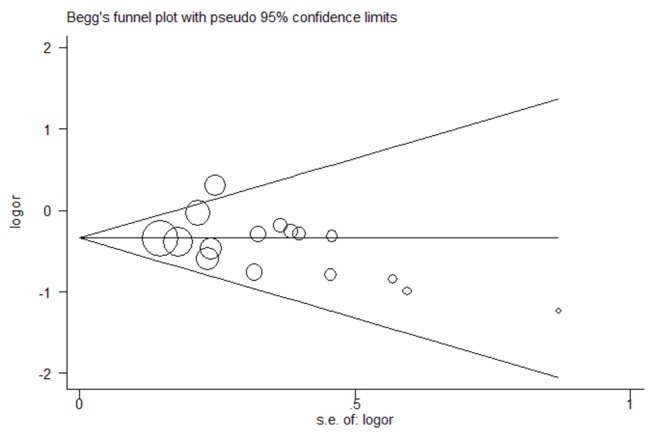
Begg’s funnel plot in the meta-analysis of the association between TNF-α 308 G/A polymorphism and psoriasis risk under the allele comparison model (A versus G).

**Figure 8:**
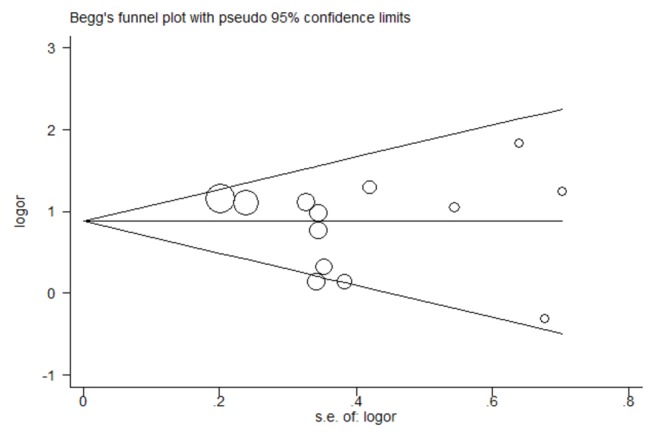
Begg’s funnel plot in the meta-analysis of the association between TNF-α 238 G/A polymorphism and psoriasis risk under the allele comparison model (A versus G).

Begg’s funnel plot was conducted to assess the publication bias of the meta-analysis, and the shape of the funnel plots seemed symmetrical in the allele comparison model (A versus G) indicating low risk of publication bias (Figure 7, and Figure 8). Besides, the Egger linear regression test also suggested there was no significant risk of publication bias (P = 0.485).

## Discussion

Psoriasis is an autoimmune and inflammatory disease, and genetic susceptibility factors also play an important role in the inflammatory and immune actions of psoriatic skin [33]. In present study, we performed a meta-analysis to comprehensively evaluate the association between TNF-α 308 G/A polymorphism and psoriasis risk and the association between TNF-α 238 G/A polymorphism and psoriasis risk. Sixteen case-control studies with a total of 2,253 psoriasis cases and 1,947 controls on TNF-α 308 G/A polymorphism [11-20,27-32] and fourteen studies on TNF-α 238 G/A polymorphism with 2,104 cases and 1,838 controls [11-17,19,28-33] were finally identified from three main databases, including Pubmed, Embase, and Web of Science. Overall, TNF-α 308 G/A polymorphism was significantly associated with decreased risk of psoriasis under three genetic comparison models when all 16 studies were pooled into the meta-analysis. Subgroup analysis by ethnicity further showed that there was a significant association between TNF-α 308 G/A polymorphism and decreased risk of psoriasis in both Caucasians and Asians. In addition, TNF-α 238 G/A polymorphism was associated with increased risk of psoriasis under three genetic models (For A versus G: fixed-effects OR 2.46, 95%CI 2.04-2.96, P < 0.001; for AG versus GG: fixed-effects OR 2.69, 95%CI 2.20-3.28, P < 0.001; for AA/AG versus GG: fixed-effects OR 2.68, 95%CI 2.20-3.26, P < 0.001). Therefore, the meta-analysis suggests that TNF-α 308 G/A polymorphism is associated with decreased risk of psoriasis, while TNF-α 238 G/A is associated with increased risk of psoriasis.

Single nucleotide conversion from guanine (G) to adenine (A) at position -308 is the most common in general populations. This transition has been shown to influence the expression of TNF-α, and a position -308 allele A is associated with a about 6 fold increased transcriptional activity and higher levels of TNF-α. TNF-α stimulates the release of interleukin 8 (IL-8) by keratinocytes and fibroblasts and transforming growth factor by keratinocytes, which may be involved in autocrine stimulation of keratinocytes proliferation in psoriatic lesions [34]. Therefore, the genetic polymorphisms in TNF-α gene can have some effects on the hosts’ susceptibility to psoriasis by the changed TNF-α expression, and TNF-α 308 G/A and 238 G/A polymorphisms are the two most commonly studied [34]. Although an association of polymorphisms in the TNF-α promoter region with psoriasis susceptibility has been reported in few previous studies, this problem remains still a matter for further research. In current study, we provided a comprehensively assessment of the association between TNF-α 308 G/A polymorphism and psoriasis risk by performing a meta-analysis of 16 eligible studies. The findings from the meta-analysis provided a strong evidence for the important role of TNF-α 308 G/A polymorphism in the development of psoriasis, and TNF-α 308 G/A mutant allele A had a protective effect on psoriasis risk.

Several limitations should be considered when interpreting the findings from this meta-analysis. Currently, there were only 3 case-control studies published to investigate the associations of TNF-α 308 G/A and 238 G/A polymorphisms with psoriasis risk in Asians, and no one studies were published to assess the association in Africans. The limited numbers of eligible studies could cause the relatively small sample size, and further resulted in poor validation and increased the risk of random error in the subgroup analysis of Asians. Therefore, more well-designed studies with large sample sizes are needed to further identify the association among Asians and Africans. Besides, the associations of TNF-α 308 G/A and 238 G/A polymorphisms with psoriasis risk in other races also need further studies.

Another limitation in the meta-analysis was that it was based on unadjusted estimates owing to the lack of adjusted estimates. Currently, several risk factors have been identified, such as family history, viral and bacterial infections, and smoking. A more precise analysis could be performed if the estimates adjusted for those possible risk factors were available. However, none of those studies reported the adjusted estimates, and to get a more precise analysis of this association, more studies with adjusted estimates are needed.

Finally, gene-gene interactions were not fully addressed in the meta-analysis for the lack of relevant data. There are several polymorphisms associated with risk of psoriasis, including signal transducer and activator of transcription 4 (STAT4), TaqI polymorphisms in vitamin D receptor (VDR) gene, interleukin-10 (IL-10) polymorphisms and LCE3B genes [35,36]. But no studies investigated the gene-gene interactions in the associations of TNF-α 308 G/A and 238 G/A polymorphisms with psoriasis risk. Future studies may further assess the possible gene-gene interactions in the association.

In summary, this meta-analysis suggests TNF-α 308 G/A polymorphism is associated with decreased risk of psoriasis, while TNF-α 238 G/A is associated with increased risk of psoriasis. More studies with adjusted estimates are needed to assess the association among Asians and Africans.

## Supporting Information

Checklist S1 **PRISMA checklist in this meta-analysis.**
(DOC)

Figure S1 **PRISMA 2009 flow diagram in this meta-analysis.**
(TIF)
