# Supplementary figures and images for: Associations between Tumor Necrosis Factor-α Polymorphisms and Risk of Psoriasis: A Meta-Analysis
Source: PLoS One. 2013 Dec 4;8(12):e68827. doi: 10.1371/journal.pone.0068827 (PMC3850909; doi:10.1371/journal.pone.0068827)

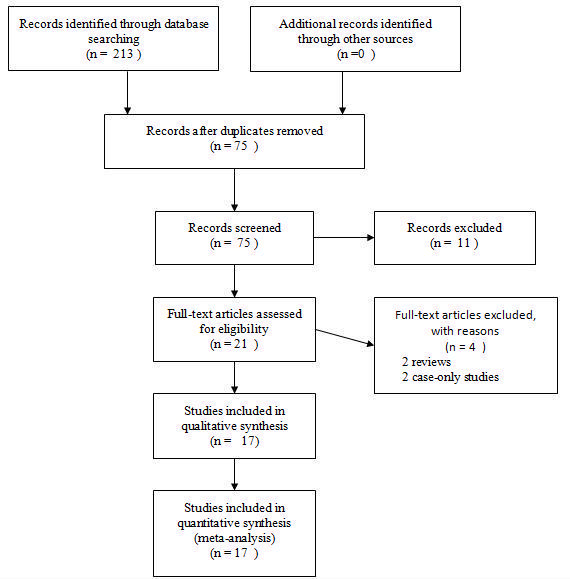

Supplement: Figure S1 — PRISMA 2009 flow diagram in this meta-analysis. (TIF) [file pone.0068827.s002.tif]
